# Response of the North Atlantic surface and intermediate ocean structure to climate warming of MIS 11

**DOI:** 10.1038/srep46192

**Published:** 2017-04-10

**Authors:** Evgenia S. Kandiano, Marcel T. J. van der Meer, Stefan Schouten, Kirsten Fahl, Jaap S. Sinninghe Damsté, Henning A. Bauch

**Affiliations:** 1 Department of Marine Microbiology and Biogeochemistry, NIOZ Netherlands Institute for Sea Research, and Utrecht University, Den Burg, NL-1790 AB, the Netherlands; 2 Department of Paleoceanography, GEOMAR Helmholtz Centre for Ocean Research Kiel, Kiel, D-24148, Germany; 3 Faculty of Geosciences, Utrecht University, Utrecht, NL-3584 CD, the Netherlands; 4 Department of Marine Geology, Alfred Wegener Institute Helmholtz Centre for Polar and Marine Research, Bremerhaven, D-27568, Germany

## Abstract

Investigating past interglacial climates not only help to understand how the climate system operates in general, it also forms a vital basis for climate predictions. We reconstructed vertical stratification changes in temperature and salinity in the North Atlantic for a period some 400 ka ago (MIS11), an interglacial time analogue of a future climate. As inferred from a unique set of biogeochemical, geochemical, and faunal data, the internal upper ocean stratification across MIS 11 shows distinct depth-dependent dynamical changes related to vertical as well as lateral shifts in the upper Atlantic meridional circulation system. Importantly, transient cold events are recognized near the end of the long phase of postglacial warming at surface, subsurface, mid, and deeper water layers. These data demonstrate that MIS 11 coolings over the North Atlantic were initially triggered by freshwater input at the surface and expansion of cold polar waters into the Subpolar Gyre. The cooling signal was then transmitted downwards into mid-water depths. Since the cold events occurred after the main deglacial phase we suggest that their cause might be related to continuous melting of the Greenland ice sheet, a mechanism that might also be relevant for the present and upcoming climate.

Recent global warming is amplified in the North Polar region through enhanced glacier melting^1^ and a reduction of the seasonal sea ice cover^2^. It is also expected that the northward propagation of oceanic and atmospheric heat and moisture from the adjacent North Atlantic region might experience significant changes due to Greenland Ice Sheet (GIS) melting^3^, sea ice loss in the Arctic^4^ and the feedback mechanisms related to these processes.

Our understanding of potential responses of the climate to these changes may be improved through investigations of past climate analogues of the upcoming period. Marine isotope stage 11 (MIS 11), a warm period which started around 425 ka, shares some aspects with our anticipated future climate, such as amplified warming in parts of the Arctic region^5^,^6^, intense melting of the GIS^7^,^8^,^9^,^10^ and, as a consequence, enhanced freshwater input into the subpolar-polar seas during this period^11^,^12^.

Although a vigorous Atlantic Meridional Overturning Circulation (AMOC) is usually inferred for MIS11^13^,^14^,^15^,^16^, interglacials as such were not free of notable climate instabilities as reflected in surface and deep waters of the North Atlantic^17^,^18^. In spite of minor variability evident in some terrestrial records^19^,^20^, the peak of MIS 11, i.e. MIS 11 sensu stricto (ss), is generally regarded as a rather stable interglacial period, at least on an interglacial time scale^13^,^16^,^21^. However, if the GIS was indeed continuously releasing meltwater during the course of this rather long interglacial^8^, there could also have been notable effects on the vertical ocean structure as well as on ocean circulation and climate. Considering the currently ongoing melting of the GIS, an investigation of such effects would help to understand the impact of future climate developments.

To unveil interglacial climate development and potential climate variability during MIS 11ss, we have reconstructed sea water properties at various depth layers combining organic and inorganic analyses of different proxy carriers. Sea surface temperatures (SSTs) were derived from the unsaturation index of long chain alkenones 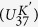
22, organic compounds produced by haptophyte algae (depth habitat 0–30 m), while temperatures of subsurface waters were reconstructed from the TEX_86_ index^23^ derived from ammonia-oxidizing *Thaumarchaeota*, which highest abundances are often found in subsurface water layers^24^. To assess temperature changes in intermediate water layers^25^ the δ^18^O composition of deep-living planktic foraminifers *Globorotalia truncatulinoides* sinistral (s) and dextral (d) were used. In addition, our temperature reconstructions are accompanied by the stable hydrogen isotopes of C_37_ alkenones^26^ (δD) and the δ^13^C of planktic foraminifer *G. bulloides* as surface salinity and subsurface water ventilation proxy, respectively. We compare these data with records which were mainly established in previous studies: SSTs based on planktic foraminiferal census counts^27^, the relative abundances of *Globigerina bulloides*^27^ and *Turborotalita quinqueloba* (new data) as environmental markers, grain counts of ice-rafted debris (IRD) as a measure for relative iceberg abundance^27^, benthic δ^13^C as a deep water ventilation proxy^15^ as well as benthic and planktic δ^18^O^27^.

## Oceanographical setting

**Figure 1:**
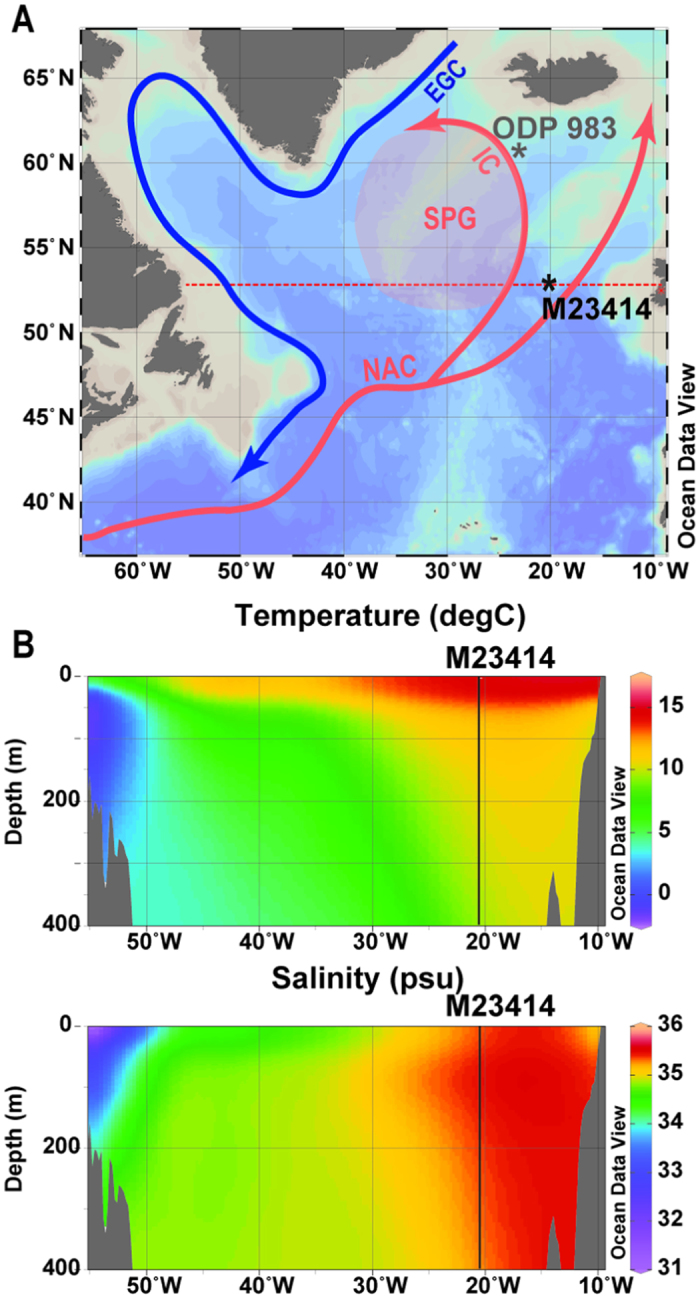
(**A**) Generalized surface ocean circulation in the North Atlantic and geographical position of investigated core M23414 (53°32′N, 20°17′W; 2196 m water depth) and reference ODP Site 983; NAC - North Atlantic Current; IC - Irminger Current; EGC - East Greenland Current; SPG – Subpolar Gyre. Red dotted line indicates transect of salinity and temperature profiles (shown on panel B). (**B**) Temperatures and salinity profiles across the NAC for the summer season, July-September. Position of core M23414 is indicated by black line. Map (**A**) and profiles (**B**) were created using the free program Ocean Data View, Version ODV 4.7.2 (available at web site odv.awi.de) and data from World Ocean Atlas (2001) (available at web site http://odv.awi.de/en/data/ocean/world_ocean_atlas_2001/).

The site where core M23414 was taken (53°32′ N, 20°17′ W; 2196 m water depth) currently underlies the western edge of the North Atlantic Current (NAC; Fig. 1), one of the most important elements of the AMOC. This location is ideal to detect lateral changes in Subpolar Gyre (SPG) configuration, since the present-day physical property differences between relatively saline and warm waters of the NAC and less saline and cold western waters of the SPG are well established. The strength of the SPG is defined by its feeding currents, the northward-flowing warm water of the NAC and the southward-flowing polar water of the East Greenland Current (EGC).

## Results and Discussion

### Climate evolution on interglacial time scale

**Figure 2:**
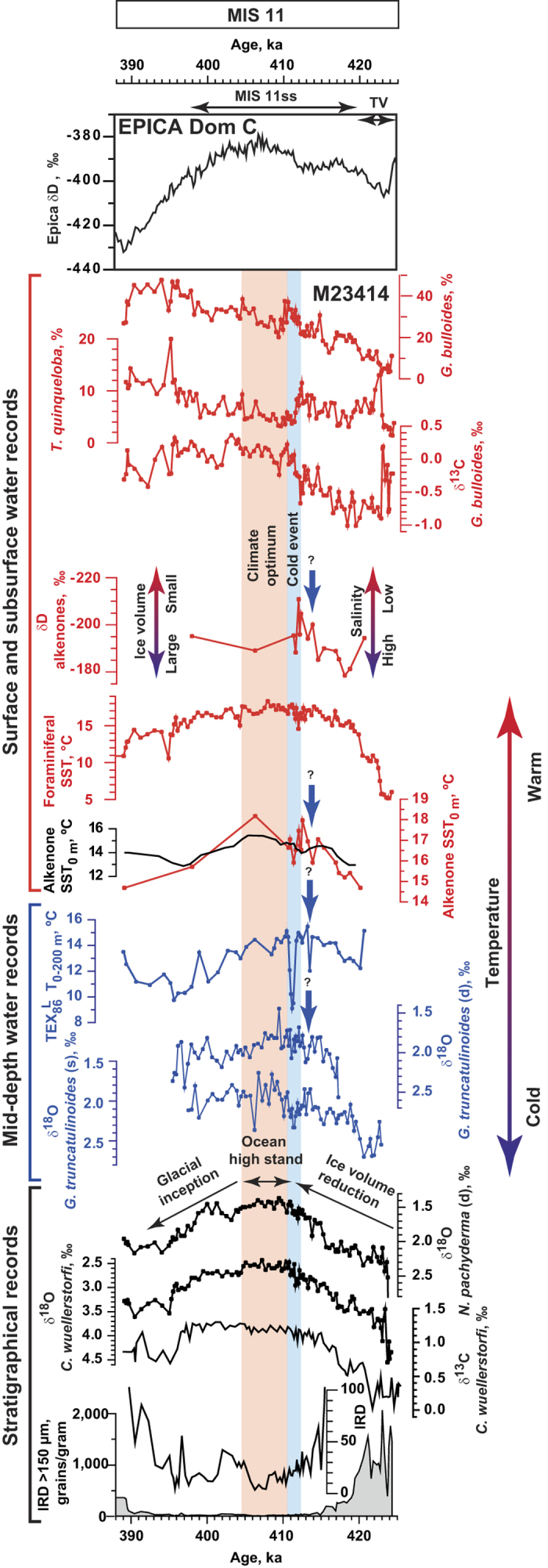
Climate related records from core M23414 in comparison to EPICA Dom C δD^31^ across MIS 11. From top to down: δD of EPICA Dom C ice core^31^. Core M23414: Relative abundance of the planktic foraminifer *G. bulloides*^27^; Relative abundance of the planktic foraminifer *T. quinqueloba*; δ^13^C of the planktic foraminifer *G. bulloides*; Alkenone δD; Summer foraminiferal SSTs reconstructed with Transfer Function Technique TFT for 10 m water depth layer^27^; 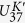 SSTs. Red line represents results from this study, black line represents the smoothed results of a previous study^30^ given for comparison (See SI); 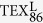 temperature reconstructions for 0–200 m water layer; δ^18^O of the planktic foraminifer *G. truncatulinoides* (dextral); δ^18^O of the planktic foraminifer *G. truncatulinoides* (sinistral); δ^18^O of the planktic foraminifer *N. pachyderma* (dextral) ^27^; δ^18^O of the benthic foraminifer C*ibicidoides wuellerstorfi*^27^; δ^13^C of the benthic foraminifer C. *wuellerstorfi*^15^ IRD on an enlarged scale^27^; IRD on a normal scale^27^. Blue bar indicates the cold event, blue arrows indicate the possible earlier cold event. MIS 11, MIS 11ss and Termination V (TV) are indicated on the top panel. TV is defined on the basis of changes in IRD content. The age models of EPICA Dom C δD and M23414 records are not tuned to each other.

According to foraminiferal SST and IRD records Termination V, the transition between full glacial conditions of MIS 12 and full interglacial conditions of MIS 11, i.e. MIS11ss, ended around 420 ka at site M23414. A sharp decrease or a complete cessation of IRD input is commonly applied in the middle latitudes of the North Atlantic to identify the main ending of terminations^28^,^29^. However, our records of foraminiferal δ^18^O as well as alkenone δD (see SI) imply that global ice volume decrease persisted well into MIS 11ss for the next 10 kyrs. Moreover, it was accompanied by a gradual interglacial temperature rise of at least 3 °C as inferred from foraminiferal, 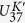, and 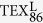 temperature records (Fig. 2). Thus, at site M23414 the start of the period of maximal temperatures occurred relatively late (410 ka) and was coincident with the time of minimal global ice volume/global sea level highstand. This time interval was therefore defined as the regional climate optimum^30^. The pattern revealed in core M23414 is in accordance with Antarctic ice-core^31^, terrestrial^7^, and marine^16^,^21^,^32^,^33^,^34^,^35^ records, which all show that the climate optimum occurred in the middle of MIS 11ss. Such a late occurrence of the climate optimum during MIS 11ss is different from the Holocene and the last interglacial when temperature rose immediately after the deglaciations. This has recently been explained by the misalignment of precession and obliquity maxima during MIS 11 that caused a gradual temperature rise at the beginning of the interglacial and an occurrence of the climate optimum during the second insolation peak within MIS 11^36^,^37^. The delay of the climate optimum in the North Atlantic during this period might additionally be aggravated by feedback mechanisms related to continuous melting of the GIS^11^,^12^ as well as enhanced freshwater export from a warm Arctic^5^. This resulted in the formation of a buoyant surface layer in the Nordic Seas^11^,^12^, which obstructed the northward Atlantic water propagation.

The benthic δ^18^O and IRD records imply that significant glacier re-advance down to sea-level happened around 396 ka, which marks the end of MIS 11ss. However, a slight change towards climate deterioration is reflected by increase of benthic and planktic δ^18^O, increase of IRD content and decrease of 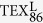 temperatures even earlier, around 405 ka, which designates the end of the climate optimum. Thus, MIS 11ss can be subdivided into three phases: a postdeglacial warming phase, which lasted ca 10 ky, the climate optimum lasting ca 5 ky, and a phase of progressive interglacial demise lasting ca 9 ky. The total estimated duration of MIS 11ss at site M23414 is around 24 ky.

### Transient cold events, their phasing, and related SPG changes

The temperature records derived from 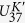 and 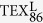 reveal an intra-interglacial transient cold event centered around 411 ka, i.e. near the very end of the prolonged phase of global ice volume decrease (Fig. 2). Interestingly, the SSTs derived from 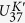 show a mere drop of ~2 °C only, whereas the 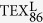 temperature reconstruction for the 0–200 m water layer indicates a much more drastic decrease of ~6 °C. The transient cold event coincided with increases in the δ^18^O of the deep dwelling planktic foraminifers *G. truncatulinoides* (s) and (d) (Fig. 2). The amplitudes of both increases reach 0.4‰ which corresponds to 1.8 °C temperature decrease when neglecting other potential factors^38^. This is in agreement with the drop in the 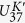 SST, but it is smaller than the one reconstructed from the 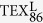. Perhaps the latter combines actual temperature changes with an effect of a vertical or seasonal migration of *Thaumarchaeota*. That might happen during interglacial cooling episodes in response to an eastward expansion of colder, more productive waters to the SPG, which then resulted in an enhanced algal production at our site. A competition for nutrients such as ammonium could have pushed the *Thaumarchaeota* to greater depths or to colder seasons^39^. Our suggestion about changes in water mass configuration is also corroborated by an increase in relative abundance of the planktic foraminiferal species *G. bulloides* from 20% before the cold event to 36% during its culmination (Fig. 2). The elevated occurrence of this species seems to be associated with the SPG, as according to the core top census data, the relative abundance of *G. bulloides* westward of site M23414 reaches up to 65%^40^ (See SI for details). After the culmination of the cold event all proxies demonstrate an abrupt return to the environmental conditions that prevailed before the cold event had started.

It is intriguing that only δ^18^O of deep-living planktic foraminiferal species *G. truncatulinoides* (s) and (d) bear a clear signature of the cold event around 411 ka, while the δ^18^O of *Neogloboquadrina pachyderma* (dextral) does not (Fig. 2). An enhanced freshwater influence on the shallower depth habitat of *N. pachyderma* (d) might be the cause for the latter observation, as this would counter-balance the temperature effect on the δ^18^O. Indeed, evidence for a freshwater input comes from a 15‰ decrease observed in alkenone δD just prior to the cold event at 412 ka (Fig. 2). Because effects of global ice volume changes can be neglected at this time, the open ocean changes in the δD of C_37_ alkenones mainly reflect salinity changes with approximately 4–5‰ of δD per salinity unit^26^ (see SI for details). Hence, a freshening of the upper water layer by ~3 salinity units seems to be a realistic estimation, suggesting a substantial freshening possibly related to ice sheet retreat and meltwater release. Other, more indirect, evidence of surface water freshening during the cold event comes from a decrease of 0.6‰ in δ^13^C of *G. bulloides*, which might reflect reduced ventilation of subsurface water due to an enhanced stratification (Fig. 2). That assumption is strengthened by a simultaneous increase in the relative abundance of the subpolar planktic foraminifer *T. quinqueloba*, a species well-adapted to colder temperatures and ice margin environments (see SI for details). The cold event at 411 ka seems to have affected the δ^18^O signature of the entire water column of the region since there is a positive response also seen in benthic δ^18^O values at this time in our core and nearby^21^. However, it is difficult to give a straightforward interpretation to this fluctuation in the benthic δ^18^O considering the absence of a response in the benthic δ^13^C record (Fig. 2). An explanation for the latter might be that the bottom of our core site is ventilated from a different source than its surface and that benthic δ^18^O represents a mixture of at least two signals: ice volume and temperature. Another cold event with similar, but less pronounced, features is revealed in our records in surface and subsurface waters around 414 ka.

Recognition of these cold events by using multiple proxies characterizing different water depths allows for reconstructing changes in the SPG during these episodes. Each of the cold events started with a freshwater injection, which changed the salinity at the surface. This freshwater input was associated with slight surface water cooling, which further evolved into substantial cooling affecting deeper water layers as inferred from the 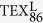 and the δ^18^O signature in *G. truncatulinoides* (s) and (d). Thus, the here identified cold events occurred in two distinct steps, implying that the first step amplified the initial cooling trend. Such a behavior of the climate system requires an involvement of feedback mechanisms as well as certain thresholds in the climate system, which allow for an abrupt amplification of cooling and a return of the system to its former state. The most important of them should be related to changes in surface water buoyancy as indicated by paleoceanographical observations and modeling experiments for both glacial and interglacial AMOC operational modes^18^,^41^,^42^,^43^,^44^,^45^.

### Supra-regional significance of MIS 11ss cold events and future climate implications

Although MIS 11ss is generally considered as a climatically stable interglacial period, at least one widespread cold event was identified in the Holsteinian terrestrial records from northern Europe. Although initially its occurrence was explained by non-climatic forcing that did not have regional significance (i.e. wildfire or a volcanic eruption^46^,^47^), more careful investigation of its evolution led to a conclusion that this cold event was climatically induced and most likely related to a short lived AMOC oscillation at the end of the global sea level rise^20^,^48^. Nevertheless, no MIS 11 paleoceanographical research has focused on abrupt cold events so far, most likely due to ambiguity of their appearance in the marine records in which they were indicated either by a single data point and/or by a single record.

**Figure 3:**
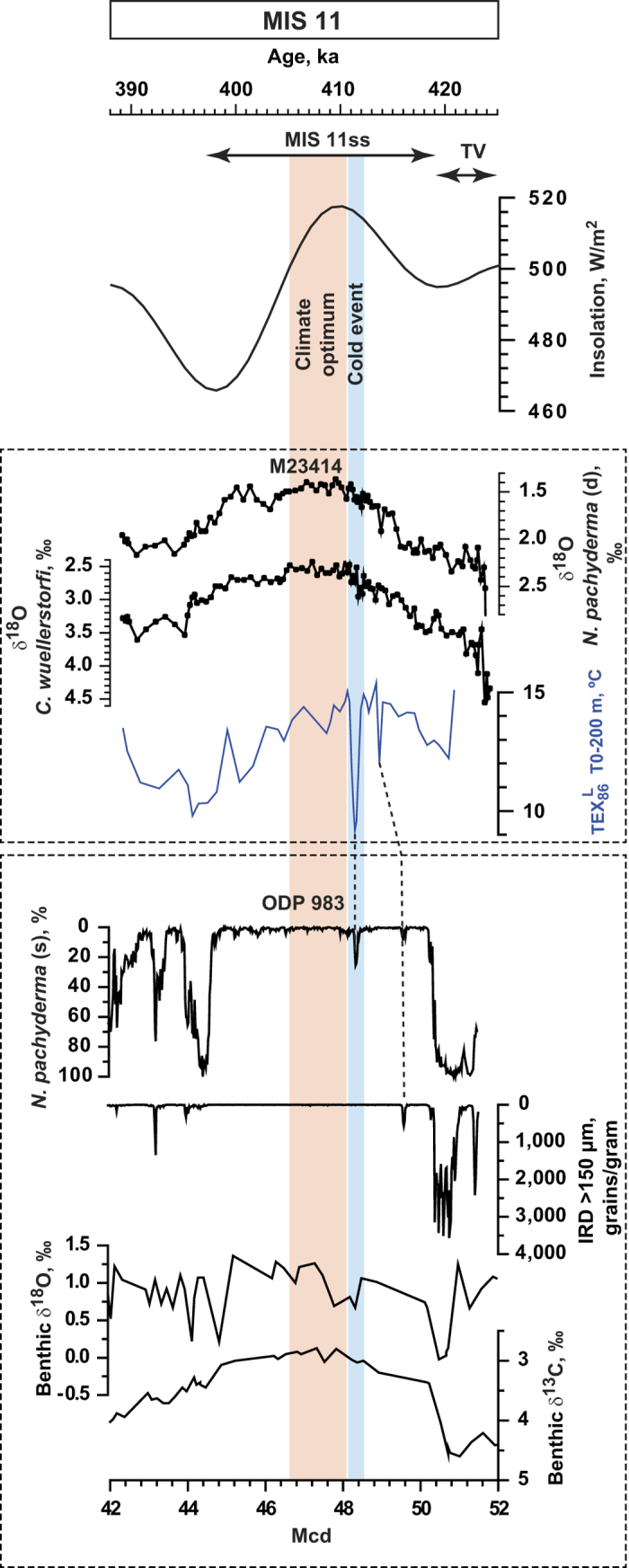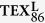 temperature reconstructions for 0–200 m water layer along with planktic and benthic δ^18^O from core M23414 compared with 21 June insolation^53^ (65°N) and climate related records from ODP Site 983: IRD^49^, relative abundance of *N. pachyderma*(s)^49^, and benthic δ^13^C and δ^18^O 50. Mcd means meter composite depth. Blue bar indicates the cold event. Dashed lines indicate a tentative correlation of the cold events between the two sites. MIS 11, MIS 11ss and Termination V (TV) are indicated on the top panel.

Further northwestward of site M23414, at ODP Site 983 (Fig. 1), two brief but significant cold events are clearly recognizable after the main period of deglacial IRD input had ceased^49^ (Fig. 3). The earlier cold event at Site 983 was associated with an IRD input, but the younger cold event, which occurred at the end of global ice volume decrease, was substantially more pronounced as seen in the increase of *N. pachyderma* (s) relative abundance. Therefore, the timing and expression of these cooling events suggest that they likely represent the cold events identified in M23414. Although at site M23414 the benthic δ^13^C record does not resolve the cold events (Fig. 2), at Site 983 the younger cold event is reflected by δ^13^C of *C. wuellerstorfi*^50^ indicating an association with the slowing down of AMOC (Fig. 3). This apparent inconsistency can be solved by considering the different deep water sources at these sites. The supra-regional character of at least the youngest of these two events is supported by 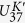 -based SST reconstructions from farther south and southeast of the SPG^33^,^34^,^35^,^51^, which all register a short climate deterioration at the end of postglacial warming, i.e. at the end of the global sea lever rise.

According to our age model the cold events at 411 and 414 ka were obviously not linked to orbital forcing^52^,^53^ (Fig. 3). Moreover, their short durations do not fit with a connection to orbital forcing or other long-term drivers of climate variability. Although we cannot rule out as a cause flood-outburst events as described for the Holocene^45^ and MIS 5e^54^, but considering the long duration of post-glacial warming during MIS 11ss and the timing of the cold events near the end of postglacial sea level rise we would rather connect them to a continue release of meltwater from the GIS. Similar polar water advances into the SPG attributed to the ice sheet retreat was inferred for MIS 5e^55^. However, during MIS 5e these advances were associated with smaller temperature amplitudes which might be explained by less intensive melting processes. This seems plausible considering that MIS 5e was also of much shorter duration in comparison to MIS 11ss^56^. In contrast, enhanced and prolonged warmth during the early phase of MIS 11ss in the North Atlantic as well as the Arctic^5^,^6^,^27^ could have accelerated GIS melting and leading to its instability^57^,^58^. This may have resulted in a rather rapid (i.e. in comparison to the elapsed part of the Holocene) deglaciation of Greenland^9^ with further development of a forest vegetation over its southern parts which is an unique environmental feature for the last million years^7^. The continuous GIS decay during MIS 11ss resulted in an extensive eastward expansion of the polar waters in the Nordic Seas^11^,^12^,^30^. This was also corroborated by icebergs persistently arriving into the central Nordic Seas during MIS 11ss^59^. It is reasonable to assume that under such conditions deep water production could occur only in the southern part of the Nordic Seas (Norwegian Sea) because the fresh and cold buoyant surface layer in the central Nordic Seas would push the Atlantic water downward preventing deep water production^11^,^12^. Considering this one can assume that AMOC, although intense on interglacial time scale^13^,^14^,^15^,^16^, could experience enhanced sensitivity to freshwater inputs into the North Atlantic eventually resulting in short-term AMOC variability. Our assumption about an influence of GIS melting on AMOC is in accordance with modern observations that have registered suppression of deep water convection (though a slight one so far) in the Labrador Sea in response to the recent acceleration of the GIS loss^60^. Our results underscore the intricate interdynamic behavior of the North Atlantic climate system. Furthermore, if the present-day rapid summer melting of the GIS continues^1^, the resulting freshening of the surface ocean may well lead to fundamental structural changes in both ocean and atmospheric circulation as reconstructed for MIS 11.

## Methods

The complete description of methods including of sample preparation for inorganic and organic analyses is provided in Supplementary Information (SI).

### TEX_86_ analyses and derived temperatures

The analysis is based on the relative abundances of isoprenoid glycerol dibiphytanyl glycerol tetraethers (GDGTs) (Schouten *et al*.^23^). For GDGT analyses, the polar fractions of the total lipid extracts were dried under N_2_, dissolved in a mixture of hexane and isopropanol (99:1, v/v) and filtered using a 0.4 μm PTFE filter. GDGT relative abundances were determined with high performance liquid chromatography/atmospheric pressure positive ionization-mass spectrometer (HPLC-MS) equipped with an auto-injector and Agilent ChemStation chromatography manager software. The core top value of 12.7 °C derived from 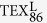
_0–200m_ matches well with the modern summer temperatures (Fig. S1). Standard deviation of replicate measurements ranges between 0–0.9 °C. The
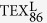
_0–200m_ temperature estimates presented in the main text were also compared to reconstructions derived from other widely used TEX_86_ calibrations (Fig. S3; all equations are placed in SI).

### Alkenone 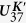 SST reconstructions

The ketone fractions from the total lipid extracts were analyzed by gas chromatography using an Agilent 6890 gas chromatograph (column CP SIL5CB, 25 mx0.32 mm, film thickness 0.12 μm) and a temperature program as follows: from 70 to 130 °C at 20 °C/min, and then to 320 °C at 4 °C/min, at which it was held isothermal for 15 min with constant pressure 70 kPa. Helium was used as a carrier gas. The SSTs were reconstructed according to ref. 22 (SST = (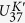 − 0.44)/0.033). 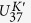
SST value reconstructed from the core tope sample (15.7 °C) is close to the modern summer SST (Fig. S1B).

### δD analysis of alkenones

The hydrogen isotopic compositions of the alkenones were determined by a GC/Thermal Conversion/isotope ratio monitoring mass spectrometer (GC-TC-irMS) using a Thermo Electron DELTA V mass spectrometer coupled to a GC-isolink. The GC was equipped with a CPsil 5 CB column, 25 meters long, 0.32 mm wide with a film thickness of 0.4 μm. The temperature program was used as follows: start at 70 °C increased with 20 °C min^−1^ to 145 °C than with 8 °C min^−1^ to 200 °C followed by 4 °C min^−1^ to 320 °C where it was kept isothermal for 25 min. Helium was used as a carrier gas with a constant flow of 1 ml/min. The H_3_^+^ correction factor was determined daily and was constant at 5.70 ± 0.03 ppm mV^−1^ for one batch of samples and 5.71 ± 0.03 ppm mV^−1^ for the majority of the samples. A standard mixture of C_16_-C_32_ *n*-alkanes with certain isotopic composition (MIX B prepared by Arndt Schimmelmann, University of Indiana) was used as a control of the systems performance and samples were only run if the average deviation of the alkanes was below 5‰ from their off-line determined value. H_2_ gas pulses with a predetermined isotopic composition were let into the ion source before and after each sample run for a standardization of the measurements. Squalane with a known δD value of −170 ± 4.0‰ was co-injected with each sample. The average of the δD_squalane_ measurements was −159.3 ± 4.2‰. The δD measurements were performed for the combined C_37:2_ and C_37:3_ alkenones. Replicate measurements were produced when possible. Standard deviation of replicate measurements ranges between 0.8‰ and 3.7‰.

### Foraminiferal and IRD counts

Foraminiferal census counts were performed using >150 μm sediment subfractions. Each sample was split by means of a microsplitter to a subsample which contained a minimum of 300 foraminiferal tests. In order to retrieve SSTs from planktic foraminiferal abundances, the Transfer Function Technique^27^ (TFT) was used. IRD were counted in >150 μm fraction^27^. In samples with a low IRD content IRD counts were performed separately from foraminiferal counts in order to achieve a better statistical accuracy of the results.

### Foraminiferal stable isotope measurements

Stable isotope measurements on planktic and benthic foraminifera were performed at the Stable Isotope Leibniz Labor (University of Kiel) using a Finnigan MAT 251 mass spectrometer with analytical accuracy of 0.07‰ and 0.03‰ for δ^18^O and δ^13^C, respectively. All measurements were calibrated on the Vienna Pee Dee Belemnite isotope scale (VPDB).

### Data deposition

All data presented in this paper are available at www.pangaea.de.

## Additional Information

**How to cite this article:** Kandiano, E. S. *et al*. Response of the North Atlantic surface and intermediate ocean structure to climate warming of MIS 11. *Sci. Rep.* **7**, 46192; doi: 10.1038/srep46192 (2017).

**Publisher's note:** Springer Nature remains neutral with regard to jurisdictional claims in published maps and institutional affiliations.

## Supplementary Material

Supplementary Information
