# Supplementary material for: Response of the North Atlantic surface and intermediate ocean structure to climate warming of MIS 11
Source: Sci Rep. 2017 Apr 10;7:46192. doi: 10.1038/srep46192 (PMC5385537; doi:10.1038/srep46192)
Supplement: Supplementary Information [file srep46192-s1.pdf]

Supplementary Information

Response of the North Atlantic surface and intermediate ocean structure to climate warming of MIS 11

Evgenia S. Kandiano[1,2*], Marcel T. J. van der Meer[1], Stefan Schouten[1,3] Kirsten Fahl[4], Jaap S. Sinninghe Damsté[1,3], and Henning A. Bauch[2,4]

[1]Department of Marine Microbiology and Biogeochemistry, NIOZ Netherlands Institute for Sea Research, and Utrecht University, Den Burg, NL-1790 AB, the Netherlands

[2]Department of Paleoceanography, GEOMAR Helmholtz Centre for Ocean Research Kiel, Kiel, D-24148, Germany

[3]Faculty of Geosciences, Utrecht University, Utrecht, NL-3584 CD, the Netherlands

[4]Department of Marine Geology, Alfred Wegener Institute Helmholtz Centre for Polar and Marine Research, Bremerhaven, D-27568, Germany

[*]To whom correspondence should be addressed. Email: ekandiano@geomar.de

Core sampling

The core section covering the full interglacial period of MIS 11ss was sampled continuously as 0.5 cm slabs while the section covering Termination V was samples as 1 cm slabs. All samples were freeze dried. For organic and inorganic analyses different sets of samples were used. All inorganic analyses were produced with 1-cm resolution while GDGT-based TEX_{86} SST reconstructions were performed in 2 cm resolution and increased to 1 cm resolution where necessary. Alkenone distributions and hydrogen isotope compositions were measured on the same sample set as GDGT, but only in those samples where sufficient amounts of alkenones were found. For comparison, all organic analyses have also been performed on the core top sample (Fig. S1A, B; See also section Methods).

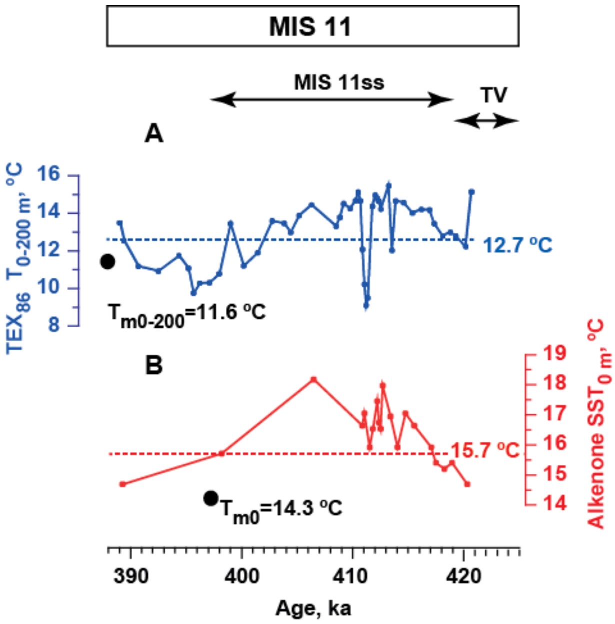

Figure S1.Temperature reconstructions during MIS 11 in comparison with modern values and temperature reconstructions in the core top sample. A: $\mathrm{TEX}^{\mathrm{L}}_{86}$ temperature reconstructions for 0-200 m water depth along with modern summer temperature of the same depth indicated by black dot (11.6 °C[26]), dashed line indicates the result of the $\mathrm{TEX}^{\mathrm{L}}_{86}$ (0-200 m) temperature reconstruction from the core top sample (12.7 °C). B: $U^{K'}_{37}$ SST reconstructions for 0 m water depth along with modern summer temperature of the same depth indicated by black dot (14.3 °C[26]). Dashed line indicates the result of the $U^{K'}_{37}$reconstruction from the core top sample (15.7 °C). MIS 11, MIS 11ss and Termination V (TV) are indicated on the top panel.

Sample preparation for inorganic analyses

Freeze dried samples were washed over 63 µm mesh-sized sieve in deionized water, dried in an oven under 40 °C. Fraction >150 µm was used.

Sample preparation for organic analyses

Total lipid extracts from freeze-dried samples were generated using Accelerated Solvent Extractor (DIONEX AS E350, 100 °C) with a mixture of dichloromethane (DCM): methanol (MeOH, 9:1 v/v). The extracts were separated into apolar, alkenone and polar fractions using Al_2O_3 columns with hexane: DCM (9:1 v/v), hexane:DCM (1:1 v/v), and DCM:MeOH (1:1 v/v), respectively.

Age model

The age model of core M23414 was established using using benthic δ^{18}O[4] (Fig. S2; The age model of a nearby ODP core 980[5] was tuned to the M2414 age model).MIS 11ss is identifiable between ~ 419 and 397 ka by a drastic decrease of the IRD content, high temperature values as well as low benthic and planktic oxygen isotope values, (Fig. 2). IRD, however, remained present during the interglacial, although in much smaller, variable amounts (Fig. 2).

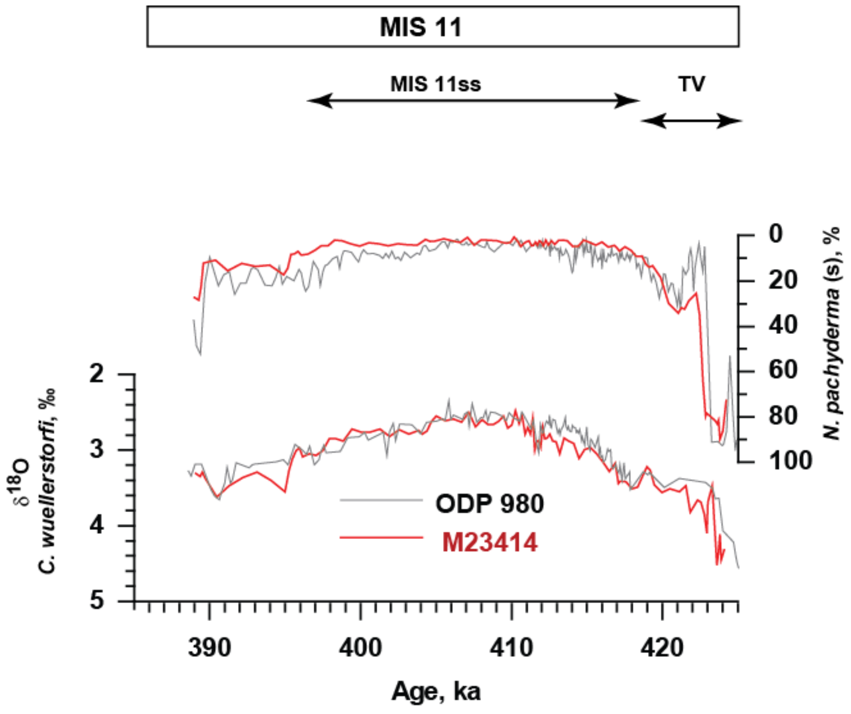

Figure S2. Relative abundance of *N. pachyderma* (s) and benthic δ^{18}O from core M23414[5] (red lines) and ODP Site 980[4] (grey lines). The age model of ODP 980 was tuned to the age model of M23414.

Comparison of TEX_{86} derived temperature estimates

In order to show that the cold event found by us is not an artifact of the calibration, we have calculated temperatures according to a variety of different widely used calibrations:

– $\mathrm{TEX}_{86}^{\mathrm{L}}$ equation[6] calibrated towards temperature in subsurface water (0-200m; $T=50.8*\log\mathrm{TEX}_{86}^{\mathrm{L}}+36.1$, where T is temperature). This record is used in the main text;

– TEX_{86}^{H} equation[7] calibrated towards temperature in subsurface water (0-200m; T=54.7*logTEX_{86}^{H} +30.7, where T is temperature);

– TEX_{86}^{H} equation[8] calibrated to SST (0 m; SST=68.4*logTEX_{86}^{H} +38.69);

– TEX_{86}^{L} equation[8] calibrated to SST (0 m; (SST=67.5*logTEX_{86}^{L}+46.9);

– Bayspar calibration[9] for TEX_{86} calibrated to SST (0 m).

Application of all calibrations yielded the same temperature trends but differed in absolute values (Fig. S3).

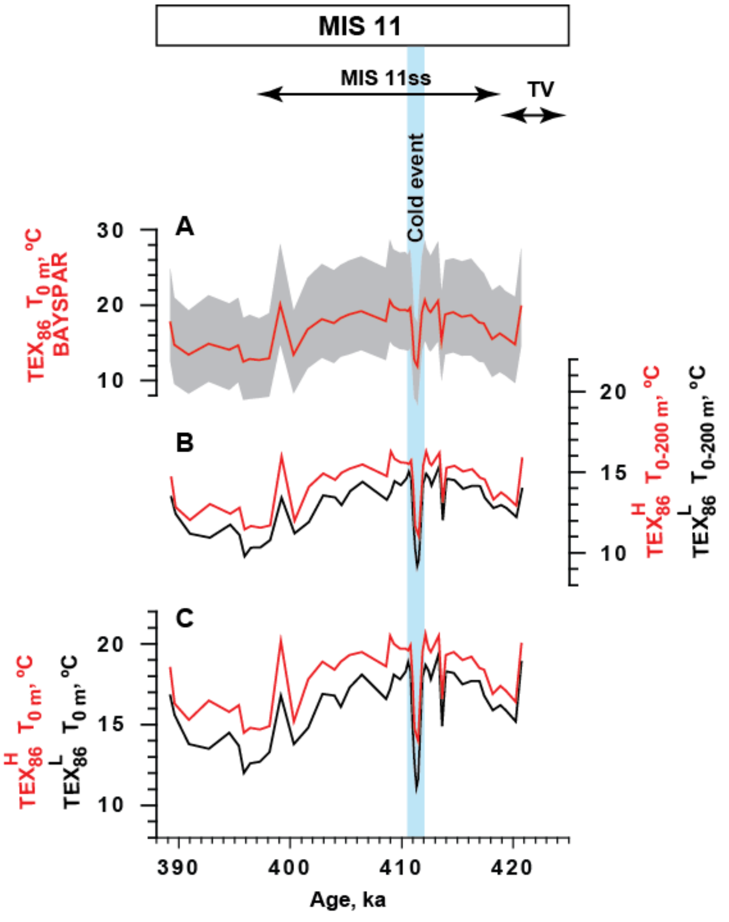

Figure S3. Comparison of TEX_{86} temperature reconstructions derived from different calibrations. Blue bar indicates cold event. MIS 11 and Termination V (TV) are indicated on the top panel. A: Bayspar surface temperature reconstructions according to ref. 9. Mean values are shown by the line while shaded area includes 90 % confidence interval; B: TEX_{86}^{L} (black line) and TEX_{86}^{H} (red line) temperature reconstructions for 0-200 m water depth layer according to ref. 6, 8; C: TEX_{86}^{L}(black line) and TEX_{86}^{H} (red line) temperature reconstructions for 0 m water depth according to ref. 8.

BIT index

The TEX_{86} proxy is known to be affected by terrestrial input which in this region will be mainly transported by ice rafted debris[10]. To constrain the effect of terrestrial input, the Branched and Isoprenoid Tetraether (BIT) indices were calculated according to ref. 11 (Fig. S4).

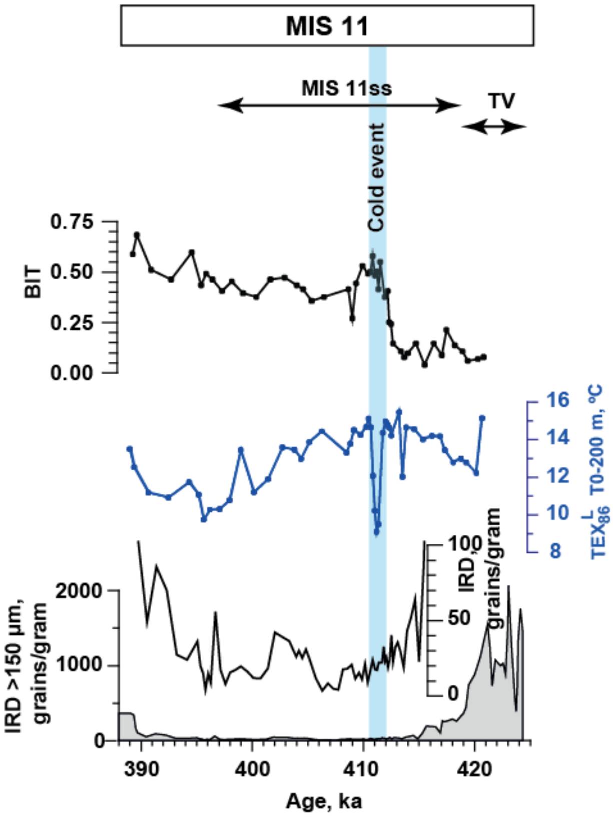

Figure S4. BIT indices in core M23414 along with $\mathrm{TEX}_{86}^{\mathrm{L}}$ temperature reconstructions for 0-200 m water depth layer and IRD[5] (note different scales for IRD on the left and right panels) across MIS 11. Blue bar indicates cold event. MIS 11 and Termination V (TV) are indicated on the top panel.

The BIT index shows relatively high values for most of MIS 11, possibly due to IRD input[10]. Alternatively, the organic matter in the sediments were exposed to oxygen and thus oxidized. Oxic degradation is known to increase the BIT index due to the better preservation of terrestrial GDGTs[12]. However, the impact of allochtonous organic matter input on the obtained temperature reconstruction is likely relatively small as we found only a low correlation between BIT and $\mathrm{TEX}_{86\ 0-200\mathrm{m}}^{\mathrm{L}}$ temperature estimates for the total MIS 11 period

(Fig. S5A) as well as for its later part, where the BIT exceed the cut off value of 0.3[13] (Fig. S5B). The absence of a strong correlation suggests no major impact of terrestrial GDGTs on the TEX_{86}, at least not for the observed cold event.

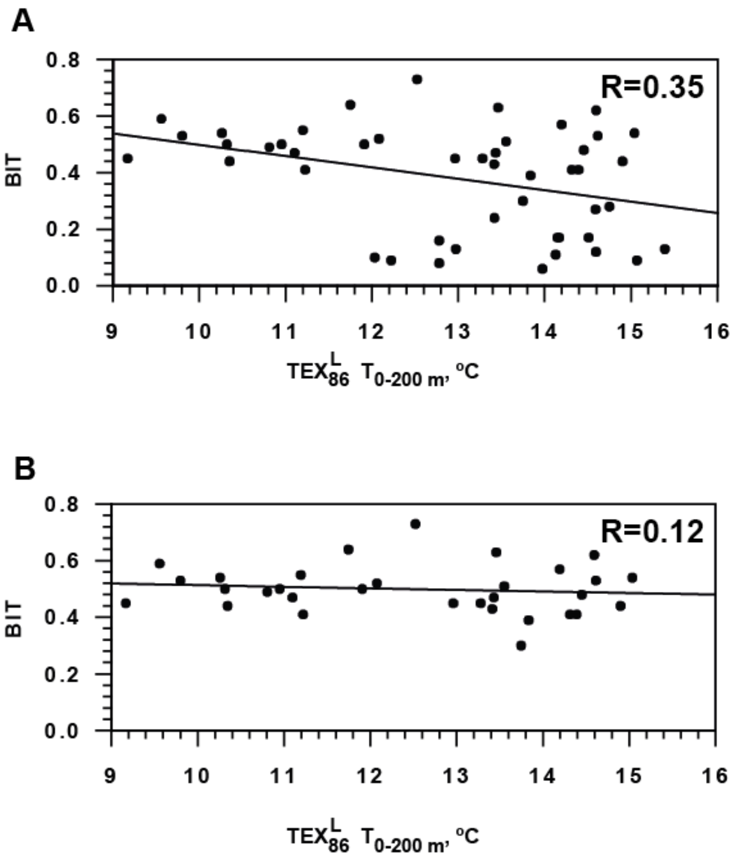

Figure S5. Correlation between $\mathrm{TEX}^{\mathrm{L}}_{86}$ temperature reconstructions for 0-200 m water depth layer and BIT indices in core M23414 across MIS 11. A: the correlation includes all $\mathrm{TEX}^{\mathrm{L}}_{86\ 0-200\mathrm{m}}$ data; B: the correlation comprises only hose$\mathrm{TEX}^{\mathrm{L}}_{86\ 0-200\mathrm{m}}$ temperature estimates in which BIT indices exceed the critical value of 0.3[13].

Comparison of the two alkenone $U^{K'}_{37}$ SST records

Comparison between our new results and those of a previously published $U^{K'}_{37}$ SST record of the same core[3] (Fig. 2, black line) displays a temperature difference of on average 2°C. This difference is likely due to the slight differences between the extraction method and instrumental conditions used in the different laboratories, in combination with very low alkenone concentrations (< 300ng/g sed). These interlaboratory differences have already

been discussed[14]. However, since we mainly focus on the trends in the temperature record, this offset is not affecting our interpretations.

Salinity reconstructions derived from δD analysis of alkenones

Culture experiments have shown that the δD value of alkenones is mainly dependent on salinity and the hydrogen isotopic composition of growth water which is also related to salinity and in a minor degree on a growth rate of alkenone producers[15,16]. A change of 4-5 ‰ in alkenone δD corresponds to a change of one salinity unit and combines both the biological response to salinity and a 1.7 ‰ δD change of the water[15,17]. In natural environments the relation between salinity and δD of water is not constant in space and time and can change with global ice volume changes due to its effect on a δD water composition[18], but also with changes in evaporation and precipitation balances. The observed intra-interglacial MIS 11ss cold event occurred at the very end of the global ice volume decrease and, therefore, the effect of ice volume changes on alkenone δD composition is most likely negligible. According to the modern distribution of δD values in the North Atlantic, the waters of the NAC have up to 6 ‰ higher δD values in comparison to the adjacent SPG waters[19]. If, by analogy to the modern state, we assume that the maximum δD depletion in surface waters at the site of M23414 associated with the MIS 11ss cold event might reach 6 ‰ due to the expansion of the western waters to the east, this would agree well with the 15 ‰ drop of alkenone δD observed during the cold event as based on the relation described in ref. 15.

Another cause of a sharp change in the alkenone δD values preceding the cold event could be a change in a species composition of alkenone producers. The Mid-Pleistocene species composition of coccolithophores at Site 980, in the close vicinity to site M23414, revealed only one dominant species *Gephyrocapsa oceanica* which produces alkenones[20]. However, it was also shown that during cold episodes the cold water indicative species *Coccolithus pelagicus* can occur in this region in relatively large amounts. Therefore this species potentially could compete with *G. oceanica* during the MIS 11ss cold event[21,21]. Although it is

thought that *C. pelagicus* does not produce alkenones, a correlation between the abundance of this species and alkenone amounts has been reported[22]. Therefore, a contribution of another species to changes in alkenone δD cannot completely be ruled out.

Ecological preferences of planktic foraminiferal species *G. bulloides and T. quinqueloba*

For this study two species with certain ecological preferences were selected: *G. bulloides* and *T. quinqueloba*. Geographical distributions of both species are given in Fig. S6. According to core top samples foraminiferal data base, both species have elevated abundances in relatively cold and fresh productive waters of the SPG situated westward from site M23414[23]. Their elevated abundances were also found at frontal zones in the Nordic seas both in surface sediments[24] and water column[25].

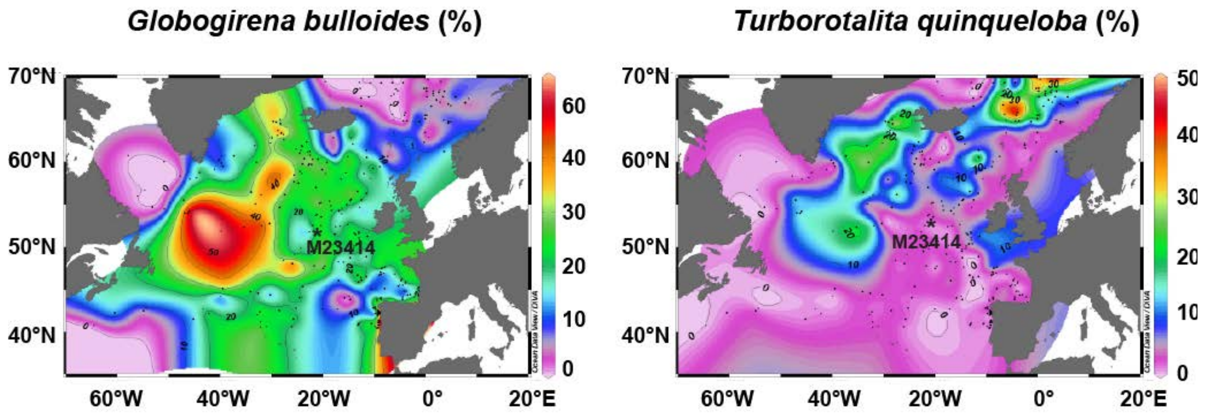

Figure S6. Geographical distribution of planktic foraminiferal species *T. quinqueloba* and *G. bulloides.* Map was created using the free program Ocean Data View, Version ODV 4.7.2 (available at web site odv.awi.de) and distribution of planktic foraminifera in core top samples according to ref. 23.

References

1 Helmke, J. P. & Bauch, H. A. Glacial-interglacial relationship between carbonate components and sediment reflectance in the North Atlantic. *Geo-Marine Letters* **21**, 16-22 (2001).

2 Helmke, J. P., Schulz, M. & Bauch, H. A. Sediment color record reveals patterns of millennial-scale climate variability over the last 500,000 years. *Quat. Res.* **57**, 16-22 (2002).

3 Kandiano, E. S. *et al.* The meridional temperature gradient in the eastern North Atlantic during MIS 11 and its link to the ocean-atmosphere system. *Palaeogeography Palaeoclimatology Palaeoecology* **333**, 24-39 (2012).

4 Kandiano, E. S. & Bauch, H. A. Phase relationship and surface water mass change in the northeast Atlantic during marine isotope stage 11 (MIS 11). *Quat. Res.* **68**, 445-45 (2007).

5 Oppo, D. W., McManus, J. F. & Cullen, J. L. Abrupt climatic events 500,000 to 340,000 years ago: Evidence from subpolar North Atlantic sediments. *Science* **279**, 1335-1338 (1998).

6 Kim, J.-H. *et al.* Holocene subsurface temperature variability in the eastern Antarctic continental margin. *Geophysical Research Letters* **39**, 10.1029/2012gl051157 (2012).

7 Kim, J.-H. *et al.* Pronounced subsurface cooling of North Atlantic waters off Northwest Africa during Dansgaard–Oeschger interstadials. *Earth and Planetary Science Letters* **339**, 95-102 (2012).

8 Kim, J.-H. *et al.* New indices and calibrations derived from the distribution of crenarchaeal isoprenoid tetraether lipids: Implications for past sea surface temperature reconstructions. *Geochimica et Cosmochimica Acta* **74**, 4639-4654 (2010).

9 Tierney, J. E. & Tingley, M. P. A Bayesian, spatially-varying calibration model for the TEX86 proxy. *Geochimica et Cosmochimica Acta* **127**, 83-106 (2014).

10 Schouten, S., Ossebaar, J., Brummer, G. J., Elderfield, H. & Damsté, J. S. S. Transport of terrestrial organic matter to the deep North Atlantic Ocean by ice rafting. *Organic Geochemistry* **38**, 1161-1168 (2007).

11 Hopmans, E. C. *et al.* A novel proxy for terrestrial organic matter in sediments based on branched and isoprenoid tetraether lipids. *Earth and Planetary Science Letters* **224**, 107-116 (2004).

12 Huguet, C. *et al.* Selective preservation of soil organic matter in oxidized marine sediments (Madeira Abyssal Plain). *Geochimica Et Cosmochimica Acta* **72**, 6061-6068 (2008).

13 Weijers, J. W. H., Schouten, S., Spaargaren, O. C. & Damsté, J. S. S. Occurrence and distribution of tetraether membrane lipids in soils: Implications for the use of the TEX86 proxy and the BIT index. *Organic Geochemistry* **37**, 1680-1693 (2006).

14 Rosell-Melé, A. *et al.* Precision of the current methods to measure the alkenone proxy U-37(K ') and absolute alkenone abundance in sediments: Results of an interlaboratory comparison study. *Geochemistry Geophysics Geosystems* **2**, 1046 (2001).

15 Schouten, S. *et al.* The effect of temperature, salinity and growth rate on the stable hydrogen isotopic composition of long chain alkenones produced by *Emiliania huxleyi* and *Gephyrocapsa oceanica*. *Biogeosciences* **3**, 113-119 (2006).

16 M'Boule, D. *et al.* Salinity dependent hydrogen isotope fractionation in alkenones produced by coastal and open ocean haptophyte algae. *Geochimica Et Cosmochimica Acta* **130**, 126-135 (2014).

17 van der Meer, M. T. J., Benthien, A., Bijma, J., Schouten, S. & Damsté, J. S. S. Alkenone distribution impacts the hydrogen isotopic composition of the C-37:2 and C-37:3 alkan-2-ones in *Emiliania huxleyi*. *Geochimica Et Cosmochimica Acta* **111**, 162-166 (2013).

18 Rohling, E. J. Paleosalinity: confidence limits and future applications. *Marine Geology* **163**, 1-11 (2000).

19 Englebrecht, A. C. & Sachs, J. P. Determination of sediment provenance at drift sites using hydrogen isotopes and unsaturation ratios in alkenones. *Geochimica Et Cosmochimica Acta* **69** (2005).

20 Marino, M., Maiorano, P. & Flower, B. P. Calcareous nannofossil changes during the Mid-Pleistocene Revolution: Paleoecologic and paleoceanographic evidence from North Atlantic Site 980/981. *Palaeogeography Palaeoclimatology Palaeoecology* **306**, 58-69 (2011).

21 Solignac, S., de Vernal, A. & Giraudeau, J. Comparison of coccolith and dinocyst assemblages in the northern North Atlantic: How well do they relate with surface hydrography? *Marine Micropaleontology* **68**, 115-135 (2008).

22 Rosell-Melé, A., Comes, P., Müller, P. J. & Ziveri, P. Alkenone fluxes and anomalous U-37(K)' values during 1989-1990 in the Northeast Atlantic (48 degrees N 21 degrees W). *Marine Chemistry* **71**, 251-264 (2000).

23 Kučera M et al. Reconstruction of sea-surface temperatures from assemblages of planktonic foraminifera: multi-technique approach based on geographically constrained calibration data sets and its application to glacial Atlantic and Pacific Oceans. *Quaternary Science Reviews* **24,** 951-998 (2005).

24 Johannessen, T., Jansen, E., Flatoy, A. & Ravelo, A. C. in *Carbon Cycling in the Glacial Ocean: Constrains of the Oceans's Role in Global Change.* (eds R. Zahn, T.F. Pedersen, M.A. Kaminski, & L. Labeyrie) 61-85 (Springer, 1994).

25 Carstens, J., Hebbeln, D. & Wefer, G. Distribution of planktic foraminifera at the ice margin in the Arctic (Fram Strait). Marine Micropaleontology **29**, 257-269 (1997).

26 Locarnini, R.A. et al. World Ocean Atlas 2013 Volume 1 Temperature. eds Levitus S NOAA Atlas NESDIS 73 40 pp. (2013).
